# 3,3′-{[(Biphenyl-2,2′-di­yl)bis­(methyl­ene)]bis­(­oxy)}bis­[*N*-(4-chloro­phen­yl)benzamide]

**DOI:** 10.1107/S160053681301009X

**Published:** 2013-05-18

**Authors:** Raj Rajadurai, Ramar Padmanabhan, Soma Sundaram Meenakshi Sundaram, Sarkkarai Ananthan

**Affiliations:** a Orchid Chemicals and Pharmaceuticals Limited, R&D Centre, Chennai 600 119, India; b Zydus Cadila, Vadodara, India; c Presidency College, Chennai 600 005, India

## Abstract

In the title compound, C_40_H_30_Cl_2_N_2_O_4_, the two benzene rings of the biphenyl unit are twisted with respect to each other, making a dihedral angle of 73.07 (4)°. The benzene rings of the benzamide groups form dihedral angles of 77.09 (5) and 55.48 (6)° with the central biphenyl moiety. In the crystal, mol­ecules are linked through N—H⋯O hydrogen bonds to form a fused *R*_2_^2^(38) ring motif which forms a supermolecular ribbon network extending along the [100] plane. In the two 4-chloro­phenyl rings, the five C atoms and their attached H atoms are disordered over two sets of sites, with site-occupancy factors of 0.657 (15):0.343 (15) and 0.509 (13):0.491 (13).

## Related literature

For the pharmacological properties of benzo[*c*]phenanthrid­ine derivatives, see: Clement *et al.* (2005 ▶); Stermitz *et al.* (1973 ▶, 1975 ▶); Fang *et al.* (1993 ▶); Suzuki *et al.* (1992 ▶); Kanzawa *et al.* (1997 ▶); Guo *et al.* (2007 ▶); Nissanka *et al.* (2001 ▶); Lenfeld *et al.* (1981 ▶); Ishikawa (2001 ▶). For the synthesis of the starting material, see: Zhang *et al.* (2008 ▶). For graph-set notation, see: Bernstein *et al.* (1995 ▶).
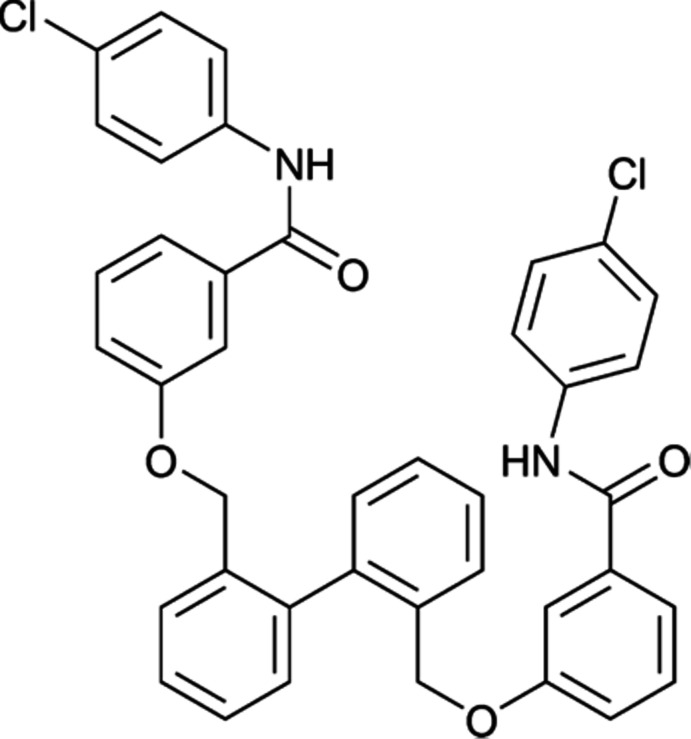


## Experimental

### 

#### Crystal data

C_40_H_30_Cl_2_N_2_O_4_
*M* *_r_* = 673.56
Triclinic, 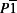

*a* = 9.4761 (2) Å
*b* = 11.9967 (3) Å
*c* = 15.9238 (4) Å
α = 75.944 (2)°
β = 86.163 (1)°
γ = 69.368 (3)°
*V* = 1643.07 (7) Å^3^
*Z* = 2
Mo *K*α radiation
μ = 0.24 mm^−1^
*T* = 296 K
0.30 × 0.25 × 0.20 mm

#### Data collection

Bruker Kappa APEXII CCD diffractometer
Absorption correction: multi-scan (*SADABS*; Bruker, 2004 ▶) *T*_min_ = 0.890, *T*_max_ = 0.953
33102 measured reflections
6937 independent reflections
4799 reflections with *I* > 2σ(*I*)
*R*_int_ = 0.032

#### Refinement

*R*[*F* ^2^ > 2σ(*F* ^2^)] = 0.039
*wR*(*F* ^2^) = 0.101
*S* = 1.02
6937 reflections
551 parameters
268 restraints
H atoms treated by a mixture of independent and constrained refinement
Δρ_max_ = 0.16 e Å^−3^
Δρ_min_ = −0.19 e Å^−3^

### 

Data collection: *APEX2* (Bruker, 2004 ▶); cell refinement: *SAINT* (Bruker, 2004 ▶); data reduction: *SAINT*; program(s) used to solve structure: *SIR92* (Altomare *et al.*, 1993 ▶); program(s) used to refine structure: *SHELXL97* (Sheldrick, 2008 ▶); molecular graphics: *ORTEP-3* (Farrugia, 2012 ▶) and *DIAMOND* (Brandenburg, 1998 ▶); software used to prepare material for publication: *PLATON* (Spek, 2009 ▶).

## Supplementary Material

Crystal structure: contains datablock(s) I, global. DOI: 10.1107/S160053681301009X/lx2280sup1.cif

Structure factors: contains datablock(s) I. DOI: 10.1107/S160053681301009X/lx2280Isup2.hkl

Supplementary material file. DOI: 10.1107/S160053681301009X/lx2280Isup3.cml

Additional supplementary materials:  crystallographic information; 3D view; checkCIF report

## Figures and Tables

**Table 1:** Hydrogen-bond geometry (Å, °)

*D*—H⋯*A*	*D*—H	H⋯*A*	*D*⋯*A*	*D*—H⋯*A*
N2—H2⋯O1^i^	0.91 (1)	2.02 (1)	2.8803 (16)	160 (2)
N1—H1⋯O4^ii^	0.90 (1)	2.05 (1)	2.9443 (16)	170 (2)

Symmetry codes: (i) 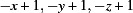; (ii) 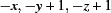.

**Fig. 1.**
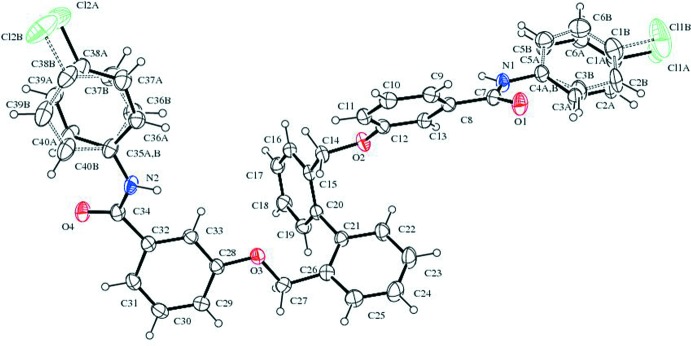
The molecular structure of the title compound with the atom numbering scheme. Displacement ellipsoids are drawn at the 30% probability level. H atoms are presented as small spheres of arbitrary radius. In the two phenyl rings of the two 4-chlorophenyl groups, the C1/C2/C3/C5/C6 atoms are disordered over two positions with site-occupancy factors, from refinement of 0.657 (15) (part A) and 0.343 (15) (part B), and the C36/C37/C38/C39/C40 atoms are disordered over two positions with site–occupancy factors, from refinement of 0.509 (13) (part A) and 0.491 (13) (part B), respectively.

**Fig. 2.**
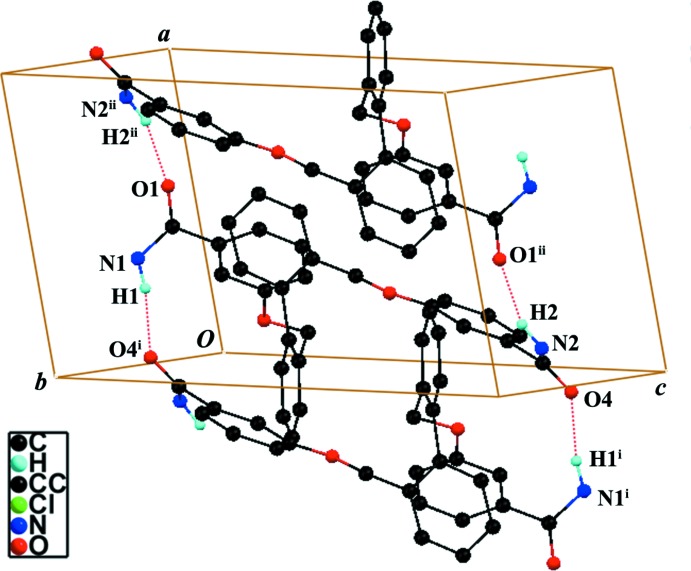
A view of N—H···O hydrogen bond interactions (dotted lines) in the crystal structure of the title compound. H atoms non–participating in hydrogen–bonding and disordered part B and D atoms were omitted for clarity. [Symmetry code: (i) - x, - y + 1, - z + 1; (ii) - x + 1, - y + 1, - z + 1.]

## supplementary crystallographic information

### Comment

Benzo[*c*]phenanthridine derivatives are a class of substances possessing a wide range of pharmacological properties. Many naturally occurring alkaloids that contain a benzo[*c*]phennthridine ring system demonstrate interesting biological ativities as mentioned in the literature (Zhang *et al.*, 2008; Clement *et al.*, 2005). Antitumor activity (Stermitz *et al.*, 1973, 1975; Fang *et al.*, 1993; Suzuki *et al.*, 1992; Kanzawa *et al.*, 1997; Guo *et al.*, 2007), antimicrobial activity (Nissanka *et al.*, 2001), anti inflammatory activity (Lenfeld *et al.*, 1981), antituberculosis activity (Ishikawa, 2001).

In the title molecule (Fig. 1), the two benzene rings of the biphenyl unit are twisted each other with a dihedral angle of 73.07 (4)°. The two phenyl rings (C8–C13 and C28–C32) of both the benzamide moiety form the dihedral angles of 77.09 (5)° and 55.48 (6)° with the central biphenyl moiety, respectively, In the two phenyl rings of the two 4–chlorophenyl groups, the five C atoms (C1/C2/C3/C5/C6) are disordered over two positions with site–occupancy factors, from refinement of 0.657 (15) (part A) and 0.343 (15) (part B), and the five C atoms (C36/C37/C38/C39/C40) are disordered over two positions with site–occupancy factors, from refinement of 0.509 (13) (part A) and 0.491 (13) (part B), respectively. In the crystal structure (Fig. 2), molecules are connected by two N—H···O hydrogen bonds (Table 1) to form fused R22 (38) ring motif which from a supermolecular ribbon network extending along the [100] plane.

### Experimental

To a solution of 3,3'-(biphenyl-2,2-diylbis(methylene))bis(oxy)dibenzoic acid (1.0 mmol) in dry DMF (10 ml), HBTU (2.5 mmol), diisopropylethyl amine (2.2 mmol) were added and stirred for 30 min. 4-Chloroaniline (3.0 mmol) was added to the reaction mixture and stirred for furher 3 h. The progress of the reaction wa monitored by HPLC. The reaction mixture was poured into ice water and filtered. The solid was washed with water folled by cold ethanol and dried under vaccum. The crude product was purified by column chromatography (SiO_2_).

### Refinement

The carbon hydrogen atoms were treated as riding atoms with a distance d(C—H) = 0.93 Å with *U*_iso_ (H) = -1.2*U*_eq_(C) [for aromatic C—H] and d(C—H) = 0.97 Å with *U*_iso_ (H) = -1.2*U*_eq_(C) [for CH_2_], respectively. The nitrogen attached H atoms identified from the difference electron density map were restrained to a distance of 0.92 (1) Å. In the molecular structure the two terminal 4–chloroaniline moieties are positionally disordered over two sites with refined occupancies set of 0.666 (15): 0.334 (15) and 0.529 (11): 0.471 (11), respectively. The positions of the atoms C4B and C35B were constrained to share the same site as that of C4A and C35A with equal atomic displacement parameters. The bond lengths of the disordered components were made similar using standard similarity restrains with suitable s.u. 0.002 Å. The ADP of the disordered compound were allowed to behave isotropically with s.u. of 0.01 followed by the appropriate rigid bond restrains.

### Crystal data

**Table:**

C_40_H_30_Cl_2_N_2_O_4_	*Z* = 2
*M**_r_* = 673.56	*F*(000) = 700
Triclinic, *P*1	*D*_x_ = 1.361 Mg m^−^^3^
Hall symbol: -P 1	Mo *K*α radiation, λ = 0.71073 Å
*a* = 9.4761 (2) Å	Cell parameters from 8220 reflections
*b* = 11.9967 (3) Å	θ = 2.3–24.7°
*c* = 15.9238 (4) Å	µ = 0.24 mm^−^^1^
α = 75.944 (2)°	*T* = 296 K
β = 86.163 (1)°	Block, colourless
γ = 69.368 (3)°	0.30 × 0.25 × 0.20 mm
*V* = 1643.07 (7) Å^3^	

### Data collection

**Table:**

Bruker Kappa APEXII CCD diffractometer	6937 independent reflections
Radiation source: fine-focus sealed tube	4799 reflections with *I* > 2σ(*I*)
Graphite monochromator	*R*_int_ = 0.032
ω and φ scan	θ_max_ = 26.7°, θ_min_ = 2.4°
Absorption correction: multi-scan (*SADABS*; Bruker, 2004)	*h* = −11→11
*T*_min_ = 0.890, *T*_max_ = 0.953	*k* = −15→15
33102 measured reflections	*l* = −20→20

### Refinement

**Table:**

Refinement on *F*^2^	Primary atom site location: structure-invariant direct methods
Least-squares matrix: full	Secondary atom site location: difference Fourier map
*R*[*F*^2^ > 2σ(*F*^2^)] = 0.039	Hydrogen site location: difference Fourier map
*wR*(*F*^2^) = 0.101	H atoms treated by a mixture of independent and constrained refinement
*S* = 1.02	*w* = 1/[σ^2^(*F*_o_^2^) + (0.0393*P*)^2^ + 0.3325*P*] where *P* = (*F*_o_^2^ + 2*F*_c_^2^)/3
6937 reflections	(Δ/σ)_max_ = 0.001
551 parameters	Δρ_max_ = 0.16 e Å^−^^3^
268 restraints	Δρ_min_ = −0.19 e Å^−^^3^

### Special details

**Table:**

Geometry. All e.s.d.'s (except the e.s.d. in the dihedral angle between two l.s. planes) are estimated using the full covariance matrix. The cell e.s.d.'s are taken into account individually in the estimation of e.s.d.'s in distances, angles and torsion angles; correlations between e.s.d.'s in cell parameters are only used when they are defined by crystal symmetry. An approximate (isotropic) treatment of cell e.s.d.'s is used for estimating e.s.d.'s involving l.s. planes.
Refinement. Refinement of *F*^2^ against ALL reflections. The weighted *R*-factor *wR* and goodness of fit *S* are based on *F*^2^, conventional *R*-factors *R* are based on *F*, with *F* set to zero for negative *F*^2^. The threshold expression of *F*^2^ > σ(*F*^2^) is used only for calculating *R*-factors(gt) *etc*. and is not relevant to the choice of reflections for refinement. *R*-factors based on *F*^2^ are statistically about twice as large as those based on *F*, and *R*- factors based on ALL data will be even larger.

### Fractional atomic coordinates and isotropic or equivalent isotropic displacement parameters (Å^2^)

**Table:**

	*x*	*y*	*z*	*U*_iso_*/*U*_eq_	Occ. (<1)
C1A	0.4646 (8)	1.2020 (6)	0.0018 (4)	0.0597 (14)	0.666 (15)
C2A	0.5814 (11)	1.0932 (7)	0.0296 (6)	0.0580 (13)	0.666 (15)
H2A	0.6728	1.0771	0.0012	0.070*	0.666 (15)
C3A	0.5602 (7)	1.0088 (9)	0.1004 (7)	0.0507 (14)	0.666 (15)
H3A	0.6365	0.9333	0.1182	0.061*	0.666 (15)
C4A	0.42764 (16)	1.03464 (14)	0.14526 (10)	0.0473 (4)	0.666 (15)
C5A	0.3087 (7)	1.1403 (5)	0.1137 (6)	0.0565 (18)	0.666 (15)
H5A	0.2151	1.1541	0.1397	0.068*	0.666 (15)
C6A	0.3294 (8)	1.2254 (7)	0.0436 (5)	0.0673 (18)	0.666 (15)
H6A	0.2513	1.2994	0.0242	0.081*	0.666 (15)
Cl1A	0.4763 (3)	1.3093 (2)	−0.09101 (16)	0.0976 (9)	0.666 (15)
C1B	0.456 (2)	1.2258 (13)	0.0167 (10)	0.080 (5)	0.334 (15)
C2B	0.575 (2)	1.1172 (15)	0.0392 (14)	0.074 (5)	0.334 (15)
H2B	0.6632	1.1063	0.0081	0.088*	0.334 (15)
C3B	0.5650 (13)	1.0243 (18)	0.1071 (15)	0.063 (5)	0.334 (15)
H3B	0.6498	0.9556	0.1269	0.076*	0.334 (15)
C4B	0.42764 (16)	1.03464 (14)	0.14526 (10)	0.0473 (4)	0.334 (15)
C5B	0.3174 (17)	1.1487 (9)	0.1283 (14)	0.062 (4)	0.334 (15)
H5B	0.2326	1.1613	0.1628	0.074*	0.334 (15)
C6B	0.3255 (19)	1.2451 (18)	0.0633 (11)	0.076 (4)	0.334 (15)
H6B	0.2466	1.3200	0.0514	0.091*	0.334 (15)
Cl1B	0.4951 (8)	1.3346 (11)	−0.0657 (11)	0.179 (3)	0.334 (15)
N1	0.40095 (14)	0.94586 (12)	0.21457 (9)	0.0492 (3)	
O1	0.63544 (12)	0.85255 (11)	0.27605 (8)	0.0621 (3)	
O2	0.17930 (12)	0.62038 (10)	0.34893 (7)	0.0500 (3)	
C7	0.50297 (16)	0.86138 (14)	0.27347 (11)	0.0449 (4)	
C8	0.44242 (15)	0.77927 (13)	0.33911 (10)	0.0411 (3)	
C9	0.49327 (16)	0.74659 (14)	0.42458 (11)	0.0459 (4)	
H9	0.5658	0.7742	0.4400	0.055*	
C10	0.43533 (18)	0.67316 (15)	0.48594 (11)	0.0481 (4)	
H10	0.4675	0.6529	0.5434	0.058*	
C11	0.32978 (17)	0.62866 (14)	0.46393 (10)	0.0457 (4)	
H11	0.2915	0.5789	0.5061	0.055*	
C12	0.28221 (16)	0.65902 (13)	0.37867 (10)	0.0405 (3)	
C13	0.33769 (16)	0.73458 (13)	0.31635 (10)	0.0415 (3)	
H13	0.3047	0.7554	0.2591	0.050*	
C14	0.13562 (18)	0.52786 (14)	0.40782 (10)	0.0466 (4)	
H14A	0.0823	0.5596	0.4559	0.056*	
H14B	0.2243	0.4577	0.4307	0.056*	
C15	0.03557 (16)	0.49016 (13)	0.36031 (9)	0.0401 (3)	
C16	−0.11978 (17)	0.54367 (14)	0.36437 (10)	0.0458 (4)	
H16	−0.1597	0.6033	0.3959	0.055*	
C17	−0.21617 (17)	0.51056 (15)	0.32282 (11)	0.0504 (4)	
H17	−0.3200	0.5477	0.3262	0.061*	
C18	−0.15817 (18)	0.42247 (16)	0.27652 (12)	0.0532 (4)	
H18	−0.2225	0.3988	0.2489	0.064*	
C19	−0.00360 (17)	0.36890 (15)	0.27094 (11)	0.0495 (4)	
H19	0.0349	0.3097	0.2389	0.059*	
C20	0.09528 (16)	0.40154 (13)	0.31206 (10)	0.0405 (3)	
C21	0.26020 (16)	0.34897 (14)	0.29640 (10)	0.0420 (4)	
C22	0.32557 (18)	0.42457 (16)	0.24000 (12)	0.0558 (4)	
H22	0.2687	0.5068	0.2191	0.067*	
C23	0.47267 (19)	0.38018 (19)	0.21453 (13)	0.0675 (5)	
H23	0.5134	0.4317	0.1757	0.081*	
C24	0.55876 (19)	0.25992 (19)	0.24657 (13)	0.0667 (5)	
H24	0.6580	0.2294	0.2294	0.080*	
C25	0.49781 (18)	0.18469 (17)	0.30407 (12)	0.0563 (4)	
H25	0.5578	0.1037	0.3268	0.068*	
C26	0.34869 (17)	0.22633 (14)	0.32927 (10)	0.0432 (4)	
C27	0.28819 (19)	0.13376 (15)	0.38488 (10)	0.0487 (4)	
H27A	0.2044	0.1309	0.3547	0.058*	
H27B	0.3662	0.0534	0.3948	0.058*	
C28	0.19174 (16)	0.07798 (13)	0.52451 (10)	0.0414 (3)	
C29	0.19905 (17)	−0.03385 (14)	0.51121 (11)	0.0480 (4)	
H29	0.2378	−0.0565	0.4602	0.058*	
C30	0.14826 (18)	−0.11133 (15)	0.57439 (12)	0.0525 (4)	
H30	0.1539	−0.1867	0.5657	0.063*	
C31	0.08990 (17)	−0.07942 (14)	0.64945 (11)	0.0476 (4)	
H31	0.0546	−0.1322	0.6908	0.057*	
C32	0.08323 (15)	0.03173 (13)	0.66397 (10)	0.0406 (3)	
C33	0.13408 (16)	0.11027 (14)	0.60071 (10)	0.0418 (3)	
H33	0.1293	0.1853	0.6097	0.050*	
C34	0.01855 (16)	0.06187 (14)	0.74681 (10)	0.0441 (4)	
C35A	0.02175 (17)	0.18781 (16)	0.84834 (10)	0.0504 (4)	0.471 (11)
C36A	0.0729 (12)	0.2814 (7)	0.8494 (8)	0.0496 (17)	0.471 (11)
H36A	0.1397	0.2992	0.8074	0.059*	0.471 (11)
C37A	0.0283 (10)	0.3494 (9)	0.9108 (5)	0.0617 (19)	0.471 (11)
H37A	0.0694	0.4086	0.9127	0.074*	0.471 (11)
C38A	−0.0781 (10)	0.3285 (7)	0.9693 (5)	0.0662 (19)	0.471 (11)
C39A	−0.1328 (8)	0.2360 (8)	0.9690 (5)	0.0677 (18)	0.471 (11)
H39A	−0.2001	0.2187	1.0110	0.081*	0.471 (11)
C40A	−0.0877 (8)	0.1692 (8)	0.9063 (4)	0.0695 (19)	0.471 (11)
H40A	−0.1310	0.1118	0.9031	0.083*	0.471 (11)
Cl2A	−0.1473 (8)	0.4066 (5)	1.0500 (4)	0.1195 (14)	0.471 (11)
C35B	0.02175 (17)	0.18781 (16)	0.84834 (10)	0.0504 (4)	0.529 (11)
C36B	0.0312 (12)	0.2991 (6)	0.8493 (7)	0.066 (3)	0.529 (11)
H36B	0.0726	0.3399	0.8027	0.079*	0.529 (11)
C37B	−0.0201 (11)	0.3507 (8)	0.9187 (5)	0.071 (2)	0.529 (11)
H37B	−0.0200	0.4285	0.9176	0.085*	0.529 (11)
C38B	−0.0716 (8)	0.2859 (8)	0.9897 (4)	0.0669 (19)	0.529 (11)
C39B	−0.0711 (9)	0.1694 (9)	0.9937 (4)	0.0815 (19)	0.529 (11)
H39B	−0.1017	0.1248	1.0432	0.098*	0.529 (11)
C40B	−0.0239 (9)	0.1210 (6)	0.9220 (2)	0.0703 (16)	0.529 (11)
H40B	−0.0228	0.0429	0.9231	0.084*	0.529 (11)
Cl2B	−0.1290 (5)	0.3534 (9)	1.0766 (3)	0.1340 (18)	0.529 (11)
N2	0.07391 (14)	0.13319 (13)	0.77786 (9)	0.0485 (3)	
O3	0.23884 (13)	0.16234 (10)	0.46603 (7)	0.0513 (3)	
O4	−0.07818 (12)	0.02208 (11)	0.78374 (8)	0.0576 (3)	
H1	0.3037 (11)	0.9519 (14)	0.2221 (10)	0.052 (5)*	
H2	0.1523 (15)	0.1504 (15)	0.7493 (10)	0.063 (5)*	

### Atomic displacement parameters (Å^2^)

**Table:**

	*U*^11^	*U*^22^	*U*^33^	*U*^12^	*U*^13^	*U*^23^
C1A	0.074 (3)	0.056 (2)	0.049 (2)	−0.031 (2)	0.0084 (18)	−0.004 (2)
C2A	0.059 (2)	0.066 (3)	0.056 (2)	−0.031 (2)	0.0106 (19)	−0.014 (2)
C3A	0.046 (2)	0.053 (2)	0.056 (3)	−0.0224 (19)	0.008 (2)	−0.010 (2)
C4A	0.0425 (8)	0.0504 (9)	0.0529 (10)	−0.0227 (7)	0.0020 (7)	−0.0097 (8)
C5A	0.043 (2)	0.062 (3)	0.056 (4)	−0.0161 (19)	0.0025 (19)	−0.003 (2)
C6A	0.071 (2)	0.055 (3)	0.060 (3)	−0.012 (2)	0.005 (2)	−0.001 (2)
Cl1A	0.1158 (14)	0.0887 (11)	0.0705 (11)	−0.0396 (10)	0.0182 (9)	0.0157 (8)
C1B	0.087 (6)	0.069 (6)	0.079 (7)	−0.035 (4)	−0.001 (5)	0.003 (5)
C2B	0.059 (5)	0.085 (7)	0.079 (8)	−0.041 (5)	0.019 (5)	−0.004 (5)
C3B	0.045 (4)	0.072 (7)	0.073 (7)	−0.029 (4)	−0.005 (4)	−0.002 (5)
C4B	0.0425 (8)	0.0504 (9)	0.0529 (10)	−0.0227 (7)	0.0020 (7)	−0.0097 (8)
C5B	0.069 (6)	0.059 (5)	0.057 (6)	−0.021 (4)	0.001 (4)	−0.016 (4)
C6B	0.076 (5)	0.067 (6)	0.074 (7)	−0.011 (4)	−0.007 (4)	−0.016 (5)
Cl1B	0.192 (4)	0.156 (4)	0.163 (6)	−0.104 (4)	−0.034 (4)	0.087 (4)
N1	0.0342 (7)	0.0530 (8)	0.0594 (9)	−0.0211 (6)	0.0044 (6)	−0.0032 (7)
O1	0.0378 (6)	0.0767 (9)	0.0713 (8)	−0.0284 (6)	0.0008 (5)	−0.0034 (7)
O2	0.0601 (7)	0.0526 (7)	0.0451 (6)	−0.0355 (6)	−0.0043 (5)	−0.0005 (5)
C7	0.0351 (8)	0.0478 (9)	0.0558 (10)	−0.0185 (7)	0.0075 (7)	−0.0146 (8)
C8	0.0329 (7)	0.0387 (8)	0.0513 (9)	−0.0116 (6)	0.0059 (6)	−0.0123 (7)
C9	0.0375 (8)	0.0451 (9)	0.0567 (10)	−0.0134 (7)	−0.0015 (7)	−0.0159 (8)
C10	0.0503 (9)	0.0474 (9)	0.0446 (9)	−0.0133 (8)	−0.0041 (7)	−0.0111 (7)
C11	0.0517 (9)	0.0412 (9)	0.0438 (9)	−0.0186 (7)	0.0028 (7)	−0.0057 (7)
C12	0.0404 (8)	0.0373 (8)	0.0453 (9)	−0.0156 (7)	0.0014 (7)	−0.0096 (7)
C13	0.0399 (8)	0.0428 (9)	0.0427 (9)	−0.0159 (7)	0.0028 (6)	−0.0100 (7)
C14	0.0551 (9)	0.0445 (9)	0.0432 (9)	−0.0262 (8)	0.0023 (7)	−0.0028 (7)
C15	0.0454 (8)	0.0376 (8)	0.0378 (8)	−0.0209 (7)	0.0026 (6)	−0.0005 (7)
C16	0.0480 (9)	0.0412 (9)	0.0455 (9)	−0.0160 (7)	0.0088 (7)	−0.0065 (7)
C17	0.0367 (8)	0.0512 (10)	0.0581 (10)	−0.0154 (7)	0.0037 (7)	−0.0040 (8)
C18	0.0429 (9)	0.0574 (11)	0.0647 (11)	−0.0241 (8)	−0.0018 (8)	−0.0132 (9)
C19	0.0451 (9)	0.0481 (9)	0.0608 (11)	−0.0192 (7)	0.0032 (8)	−0.0186 (8)
C20	0.0398 (8)	0.0382 (8)	0.0428 (8)	−0.0175 (7)	0.0018 (6)	−0.0027 (7)
C21	0.0373 (8)	0.0452 (9)	0.0460 (9)	−0.0183 (7)	0.0018 (6)	−0.0094 (7)
C22	0.0447 (9)	0.0520 (10)	0.0667 (12)	−0.0202 (8)	0.0037 (8)	−0.0024 (9)
C23	0.0473 (10)	0.0727 (13)	0.0778 (13)	−0.0291 (10)	0.0091 (9)	0.0014 (11)
C24	0.0367 (9)	0.0782 (14)	0.0801 (14)	−0.0196 (9)	0.0071 (9)	−0.0111 (11)
C25	0.0426 (9)	0.0555 (11)	0.0629 (11)	−0.0100 (8)	−0.0042 (8)	−0.0088 (9)
C26	0.0434 (8)	0.0478 (9)	0.0397 (8)	−0.0178 (7)	−0.0021 (7)	−0.0093 (7)
C27	0.0538 (9)	0.0469 (9)	0.0446 (9)	−0.0168 (8)	0.0038 (7)	−0.0109 (7)
C28	0.0392 (8)	0.0378 (8)	0.0447 (9)	−0.0134 (7)	−0.0010 (7)	−0.0044 (7)
C29	0.0498 (9)	0.0402 (9)	0.0524 (10)	−0.0128 (7)	0.0020 (7)	−0.0122 (8)
C30	0.0552 (10)	0.0368 (9)	0.0663 (12)	−0.0172 (8)	−0.0006 (8)	−0.0112 (8)
C31	0.0414 (8)	0.0424 (9)	0.0573 (10)	−0.0181 (7)	−0.0007 (7)	−0.0026 (8)
C32	0.0303 (7)	0.0422 (9)	0.0480 (9)	−0.0133 (6)	−0.0013 (6)	−0.0066 (7)
C33	0.0403 (8)	0.0380 (8)	0.0485 (9)	−0.0150 (7)	0.0024 (7)	−0.0111 (7)
C34	0.0314 (7)	0.0475 (9)	0.0501 (9)	−0.0147 (7)	0.0008 (7)	−0.0037 (7)
C35A	0.0398 (8)	0.0713 (12)	0.0436 (9)	−0.0242 (8)	0.0049 (7)	−0.0136 (8)
C36A	0.053 (4)	0.052 (3)	0.042 (3)	−0.018 (3)	0.001 (3)	−0.009 (3)
C37A	0.067 (5)	0.071 (4)	0.051 (3)	−0.031 (3)	0.004 (3)	−0.012 (3)
C38A	0.082 (4)	0.069 (4)	0.051 (4)	−0.032 (3)	0.010 (3)	−0.015 (3)
C39A	0.064 (3)	0.087 (4)	0.068 (4)	−0.039 (3)	0.033 (3)	−0.038 (3)
C40A	0.048 (3)	0.107 (4)	0.082 (3)	−0.049 (3)	0.033 (3)	−0.051 (3)
Cl2A	0.166 (3)	0.135 (3)	0.091 (2)	−0.068 (2)	0.039 (2)	−0.0722 (19)
C35B	0.0398 (8)	0.0713 (12)	0.0436 (9)	−0.0242 (8)	0.0049 (7)	−0.0136 (8)
C36B	0.081 (6)	0.058 (3)	0.043 (3)	−0.012 (3)	0.001 (3)	−0.003 (2)
C37B	0.077 (5)	0.077 (3)	0.058 (3)	−0.019 (3)	0.005 (3)	−0.026 (3)
C38B	0.060 (3)	0.101 (5)	0.048 (3)	−0.032 (4)	0.009 (2)	−0.029 (3)
C39B	0.080 (4)	0.123 (5)	0.058 (3)	−0.056 (4)	0.023 (3)	−0.025 (3)
C40B	0.062 (4)	0.105 (4)	0.063 (3)	−0.049 (3)	0.022 (2)	−0.032 (2)
Cl2B	0.1161 (14)	0.217 (5)	0.083 (2)	−0.043 (3)	0.0195 (15)	−0.090 (3)
N2	0.0418 (7)	0.0659 (9)	0.0470 (8)	−0.0310 (7)	0.0128 (6)	−0.0149 (7)
O3	0.0683 (7)	0.0463 (6)	0.0445 (6)	−0.0270 (6)	0.0114 (5)	−0.0121 (5)
O4	0.0440 (6)	0.0703 (8)	0.0654 (8)	−0.0330 (6)	0.0127 (5)	−0.0118 (6)

### Geometric parameters (Å, º)

**Table:**

C1A—C6A	1.373 (2)	C21—C22	1.392 (2)
C1A—C2A	1.375 (2)	C21—C26	1.399 (2)
C1A—Cl1A	1.735 (3)	C22—C23	1.377 (2)
C2A—C3A	1.376 (2)	C22—H22	0.9300
C2A—H2A	0.9300	C23—C24	1.370 (3)
C3A—C4A	1.378 (2)	C23—H23	0.9300
C3A—H3A	0.9300	C24—C25	1.372 (2)
C4A—C5A	1.3747 (19)	C24—H24	0.9300
C5A—C6A	1.3738 (19)	C25—C26	1.390 (2)
C5A—H5A	0.9300	C25—H25	0.9300
C6A—H6A	0.9300	C26—C27	1.500 (2)
C1B—C6B	1.373 (2)	C27—O3	1.4239 (18)
C1B—C2B	1.375 (2)	C27—H27A	0.9700
C1B—Cl1B	1.735 (3)	C27—H27B	0.9700
C2B—C3B	1.376 (2)	C28—O3	1.3767 (17)
C2B—H2B	0.9300	C28—C33	1.383 (2)
C3B—H3B	0.9300	C28—C29	1.386 (2)
C5B—C6B	1.373 (2)	C29—C30	1.380 (2)
C5B—H5B	0.9300	C29—H29	0.9300
C6B—H6B	0.9300	C30—C31	1.366 (2)
N1—C7	1.346 (2)	C30—H30	0.9300
N1—H1	0.901 (9)	C31—C32	1.387 (2)
O1—C7	1.2247 (17)	C31—H31	0.9300
O2—C12	1.3702 (17)	C32—C33	1.389 (2)
O2—C14	1.4314 (17)	C32—C34	1.488 (2)
C7—C8	1.487 (2)	C33—H33	0.9300
C8—C13	1.386 (2)	C34—O4	1.2303 (17)
C8—C9	1.391 (2)	C34—N2	1.345 (2)
C9—C10	1.372 (2)	C35A—C36A	1.374 (2)
C9—H9	0.9300	C35A—C40A	1.383 (2)
C10—C11	1.386 (2)	C35A—N2	1.410 (2)
C10—H10	0.9300	C36A—C37A	1.374 (2)
C11—C12	1.380 (2)	C36A—H36A	0.9300
C11—H11	0.9300	C37A—C38A	1.373 (2)
C12—C13	1.383 (2)	C37A—H37A	0.9300
C13—H13	0.9300	C38A—C39A	1.382 (2)
C14—C15	1.497 (2)	C38A—Cl2A	1.731 (3)
C14—H14A	0.9700	C39A—C40A	1.383 (2)
C14—H14B	0.9700	C39A—H39A	0.9300
C15—C16	1.387 (2)	C40A—H40A	0.9300
C15—C20	1.398 (2)	C36B—C37B	1.374 (2)
C16—C17	1.376 (2)	C36B—H36B	0.9300
C16—H16	0.9300	C37B—C38B	1.373 (2)
C17—C18	1.370 (2)	C37B—H37B	0.9300
C17—H17	0.9300	C38B—C39B	1.382 (2)
C18—C19	1.383 (2)	C38B—Cl2B	1.732 (3)
C18—H18	0.9300	C39B—C40B	1.383 (2)
C19—C20	1.388 (2)	C39B—H39B	0.9300
C19—H19	0.9300	C40B—H40B	0.9300
C20—C21	1.494 (2)	N2—H2	0.905 (9)
			
C6A—C1A—C2A	120.6 (5)	C26—C21—C20	123.74 (13)
C6A—C1A—Cl1A	117.3 (5)	C23—C22—C21	121.43 (16)
C2A—C1A—Cl1A	121.9 (5)	C23—C22—H22	119.3
C1A—C2A—C3A	118.6 (8)	C21—C22—H22	119.3
C1A—C2A—H2A	120.7	C24—C23—C22	119.83 (16)
C3A—C2A—H2A	120.7	C24—C23—H23	120.1
C2A—C3A—C4A	121.0 (8)	C22—C23—H23	120.1
C2A—C3A—H3A	119.5	C23—C24—C25	119.67 (16)
C4A—C3A—H3A	119.5	C23—C24—H24	120.2
C5A—C4A—C3A	119.6 (5)	C25—C24—H24	120.2
C6A—C5A—C4A	119.4 (6)	C24—C25—C26	121.69 (16)
C6A—C5A—H5A	120.3	C24—C25—H25	119.2
C4A—C5A—H5A	120.3	C26—C25—H25	119.2
C1A—C6A—C5A	120.4 (7)	C25—C26—C21	118.80 (14)
C1A—C6A—H6A	119.8	C25—C26—C27	117.51 (15)
C5A—C6A—H6A	119.8	C21—C26—C27	123.49 (14)
C6B—C1B—C2B	120.4 (12)	O3—C27—C26	111.81 (13)
C6B—C1B—Cl1B	125.4 (11)	O3—C27—H27A	109.3
C2B—C1B—Cl1B	113.8 (11)	C26—C27—H27A	109.3
C1B—C2B—C3B	121.1 (16)	O3—C27—H27B	109.3
C1B—C2B—H2B	119.5	C26—C27—H27B	109.3
C3B—C2B—H2B	119.5	H27A—C27—H27B	107.9
C2B—C3B—H3B	120.5	O3—C28—C33	116.01 (13)
C6B—C5B—H5B	118.2	O3—C28—C29	124.31 (14)
C1B—C6B—C5B	116.9 (16)	C33—C28—C29	119.68 (14)
C1B—C6B—H6B	121.5	C30—C29—C28	119.35 (15)
C5B—C6B—H6B	121.5	C30—C29—H29	120.3
C7—N1—H1	117.6 (10)	C28—C29—H29	120.3
C12—O2—C14	117.22 (11)	C31—C30—C29	121.23 (15)
O1—C7—N1	124.04 (14)	C31—C30—H30	119.4
O1—C7—C8	121.00 (15)	C29—C30—H30	119.4
N1—C7—C8	114.94 (12)	C30—C31—C32	120.03 (14)
C13—C8—C9	119.87 (14)	C30—C31—H31	120.0
C13—C8—C7	121.17 (14)	C32—C31—H31	120.0
C9—C8—C7	118.96 (13)	C31—C32—C33	119.10 (15)
C10—C9—C8	119.32 (14)	C31—C32—C34	117.79 (13)
C10—C9—H9	120.3	C33—C32—C34	123.10 (14)
C8—C9—H9	120.3	C28—C33—C32	120.60 (14)
C9—C10—C11	121.23 (15)	C28—C33—H33	119.7
C9—C10—H10	119.4	C32—C33—H33	119.7
C11—C10—H10	119.4	O4—C34—N2	123.28 (15)
C12—C11—C10	119.25 (14)	O4—C34—C32	121.08 (14)
C12—C11—H11	120.4	N2—C34—C32	115.63 (12)
C10—C11—H11	120.4	C36A—C35A—C40A	118.5 (5)
O2—C12—C11	124.68 (13)	C36A—C35A—N2	113.6 (4)
O2—C12—C13	115.13 (13)	C40A—C35A—N2	127.2 (2)
C11—C12—C13	120.18 (14)	C37A—C36A—C35A	122.0 (8)
C12—C13—C8	120.12 (14)	C37A—C36A—H36A	119.0
C12—C13—H13	119.9	C35A—C36A—H36A	119.0
C8—C13—H13	119.9	C38A—C37A—C36A	118.9 (9)
O2—C14—C15	108.62 (12)	C38A—C37A—H37A	120.5
O2—C14—H14A	110.0	C36A—C37A—H37A	120.5
C15—C14—H14A	110.0	C37A—C38A—C39A	120.2 (6)
O2—C14—H14B	110.0	C37A—C38A—Cl2A	124.7 (6)
C15—C14—H14B	110.0	C39A—C38A—Cl2A	115.0 (5)
H14A—C14—H14B	108.3	C38A—C39A—C40A	120.0 (5)
C16—C15—C20	119.12 (14)	C38A—C39A—H39A	120.0
C16—C15—C14	119.48 (14)	C40A—C39A—H39A	120.0
C20—C15—C14	121.40 (13)	C39A—C40A—C35A	120.0 (4)
C17—C16—C15	121.51 (15)	C39A—C40A—H40A	120.0
C17—C16—H16	119.2	C35A—C40A—H40A	120.0
C15—C16—H16	119.2	C37B—C36B—H36B	119.8
C18—C17—C16	119.57 (15)	C38B—C37B—C36B	119.2 (8)
C18—C17—H17	120.2	C38B—C37B—H37B	120.4
C16—C17—H17	120.2	C36B—C37B—H37B	120.4
C17—C18—C19	119.81 (15)	C37B—C38B—C39B	121.8 (5)
C17—C18—H18	120.1	C37B—C38B—Cl2B	117.1 (5)
C19—C18—H18	120.1	C39B—C38B—Cl2B	121.0 (4)
C18—C19—C20	121.42 (15)	C38B—C39B—C40B	118.0 (4)
C18—C19—H19	119.3	C38B—C39B—H39B	121.0
C20—C19—H19	119.3	C40B—C39B—H39B	121.0
C19—C20—C15	118.57 (13)	C39B—C40B—H40B	119.6
C19—C20—C21	118.54 (14)	C34—N2—C35A	127.85 (13)
C15—C20—C21	122.66 (13)	C34—N2—H2	116.5 (11)
C22—C21—C26	118.54 (14)	C35A—N2—H2	115.6 (11)
C22—C21—C20	117.49 (14)	C28—O3—C27	116.35 (12)
			
C6A—C1A—C2A—C3A	−0.4 (16)	C20—C21—C22—C23	−172.93 (17)
Cl1A—C1A—C2A—C3A	−175.5 (9)	C21—C22—C23—C24	−1.6 (3)
C1A—C2A—C3A—C4A	−3.1 (18)	C22—C23—C24—C25	−0.2 (3)
C2A—C3A—C4A—C5A	7.1 (16)	C23—C24—C25—C26	1.8 (3)
C3A—C4A—C5A—C6A	−7.5 (13)	C24—C25—C26—C21	−1.5 (3)
C2A—C1A—C6A—C5A	−0.1 (15)	C24—C25—C26—C27	173.61 (16)
Cl1A—C1A—C6A—C5A	175.2 (8)	C22—C21—C26—C25	−0.3 (2)
C4A—C5A—C6A—C1A	4.1 (14)	C20—C21—C26—C25	174.13 (15)
C6B—C1B—C2B—C3B	2 (3)	C22—C21—C26—C27	−175.11 (15)
Cl1B—C1B—C2B—C3B	176 (2)	C20—C21—C26—C27	−0.7 (2)
C2B—C1B—C6B—C5B	−4 (3)	C25—C26—C27—O3	119.95 (16)
Cl1B—C1B—C6B—C5B	−177.7 (18)	C21—C26—C27—O3	−65.20 (19)
O1—C7—C8—C13	−141.98 (16)	O3—C28—C29—C30	−179.90 (14)
N1—C7—C8—C13	39.9 (2)	C33—C28—C29—C30	0.1 (2)
O1—C7—C8—C9	37.6 (2)	C28—C29—C30—C31	0.5 (2)
N1—C7—C8—C9	−140.53 (15)	C29—C30—C31—C32	−1.1 (2)
C13—C8—C9—C10	−2.0 (2)	C30—C31—C32—C33	1.1 (2)
C7—C8—C9—C10	178.49 (14)	C30—C31—C32—C34	179.99 (14)
C8—C9—C10—C11	1.5 (2)	O3—C28—C33—C32	179.91 (13)
C9—C10—C11—C12	−0.1 (2)	C29—C28—C33—C32	−0.1 (2)
C14—O2—C12—C11	−9.8 (2)	C31—C32—C33—C28	−0.5 (2)
C14—O2—C12—C13	170.86 (13)	C34—C32—C33—C28	−179.32 (13)
C10—C11—C12—O2	179.68 (14)	C31—C32—C34—O4	−29.4 (2)
C10—C11—C12—C13	−1.0 (2)	C33—C32—C34—O4	149.42 (15)
O2—C12—C13—C8	179.96 (13)	C31—C32—C34—N2	149.94 (14)
C11—C12—C13—C8	0.6 (2)	C33—C32—C34—N2	−31.2 (2)
C9—C8—C13—C12	0.9 (2)	C40A—C35A—C36A—C37A	5.7 (13)
C7—C8—C13—C12	−179.55 (14)	N2—C35A—C36A—C37A	176.8 (8)
C12—O2—C14—C15	−174.41 (13)	C35A—C36A—C37A—C38A	−4.3 (15)
O2—C14—C15—C16	−95.68 (16)	C36A—C37A—C38A—C39A	3.4 (13)
O2—C14—C15—C20	84.61 (17)	C36A—C37A—C38A—Cl2A	−179.5 (9)
C20—C15—C16—C17	0.7 (2)	C37A—C38A—C39A—C40A	−4.0 (12)
C14—C15—C16—C17	−179.01 (14)	Cl2A—C38A—C39A—C40A	178.6 (5)
C15—C16—C17—C18	0.2 (2)	C38A—C39A—C40A—C35A	5.5 (9)
C16—C17—C18—C19	−0.9 (2)	C36A—C35A—C40A—C39A	−6.2 (9)
C17—C18—C19—C20	0.6 (3)	N2—C35A—C40A—C39A	−176.0 (4)
C18—C19—C20—C15	0.3 (2)	C36B—C37B—C38B—C39B	1.0 (12)
C18—C19—C20—C21	−174.20 (15)	C36B—C37B—C38B—Cl2B	177.9 (7)
C16—C15—C20—C19	−1.0 (2)	C37B—C38B—C39B—C40B	−3.2 (9)
C14—C15—C20—C19	178.72 (13)	Cl2B—C38B—C39B—C40B	−180.0 (5)
C16—C15—C20—C21	173.33 (13)	O4—C34—N2—C35A	−9.0 (3)
C14—C15—C20—C21	−7.0 (2)	C32—C34—N2—C35A	171.68 (15)
C19—C20—C21—C22	102.90 (18)	C36A—C35A—N2—C34	−162.8 (6)
C15—C20—C21—C22	−71.4 (2)	C40A—C35A—N2—C34	7.4 (6)
C19—C20—C21—C26	−71.6 (2)	C33—C28—O3—C27	−175.62 (13)
C15—C20—C21—C26	114.09 (17)	C29—C28—O3—C27	4.4 (2)
C26—C21—C22—C23	1.9 (3)	C26—C27—O3—C28	−175.29 (12)

### Hydrogen-bond geometry (Å, º)

**Table:**

*D*—H···*A*	*D*—H	H···*A*	*D*···*A*	*D*—H···*A*
N2—H2···O1^i^	0.91 (1)	2.02 (1)	2.8803 (16)	160 (2)
N1—H1···O4^ii^	0.90 (1)	2.05 (1)	2.9443 (16)	170 (2)

Symmetry codes: (i) −*x*+1, −*y*+1, −*z*+1; (ii) −*x*, −*y*+1, −*z*+1.
